# The umbilical canal revisited: historical anatomy and radiologic implications of a commonly misnamed CT finding

**DOI:** 10.1007/s00261-026-05440-1

**Published:** 2026-03-09

**Authors:** André Vaz, Vinícius Cardoso Serra, Camila Pietroski Reifegerste

**Affiliations:** 1 Instituto do Coração do Hospital de Clínicas da FMUSP, Universidade de São Paulo, Sao Paulo, Brazil; 2 Beneficência Portuguesa de São Paulo, Sao Paulo, Brazil

**Keywords:** Umbilical hernia, Abdominal wall, Peritoneum, Computed tomography, Cross-sectional anatomy

## Abstract

**Background:**

The increasing use of cross-sectional imaging—particularly abdominal computed tomography (CT)—together with the global rise in obesity has brought renewed attention to a fat-containing structure posterior to the umbilical scar that is frequently labeled as an “umbilical hernia” in radiology reports. Historical anatomical literature, however, describes this configuration as the *umbilical canal*, a variable, habitus-dependent space between the posterior surface of the linea alba and a reinforcing lamella of the transversalis fascia, often filled with preperitoneal fat and containing embryologic remnants of the umbilical vessels.

**Purpose:**

To revisit the anatomical foundations of the umbilical canal, correlate classical descriptions with modern CT appearances, and examine how contemporary surgical risk metrics may be inappropriately extrapolated to this structure.

**Discussion:**

Classical anatomical and surgical literature—from Scarpa, Richet, Gauderon, and Cullen to early twentieth-century surgical pathology—consistently distinguishes true umbilical hernia, defined by a peritoneal-lined sac, a fascial neck, and visceral content, from preperitoneal fat protrusions within the linea alba. Modern CT imaging frequently demonstrates a funnel-shaped, fat-filled tract posterior to the umbilical scar that corresponds to the historically described umbilical canal rather than a true hernia. Because this configuration lacks a peritoneal sac and a discrete fascial neck, applying hernia-based morphologic risk metrics may lead to diagnostic ambiguity.

**Conclusion:**

Recognizing the umbilical canal as a distinct anatomical configuration rather than a true umbilical hernia may improve terminological precision in radiology reporting, enhance interdisciplinary communication, and help avoid potential overdiagnosis and unnecessary surgical referral.

## Introduction

**Fig. 1:**
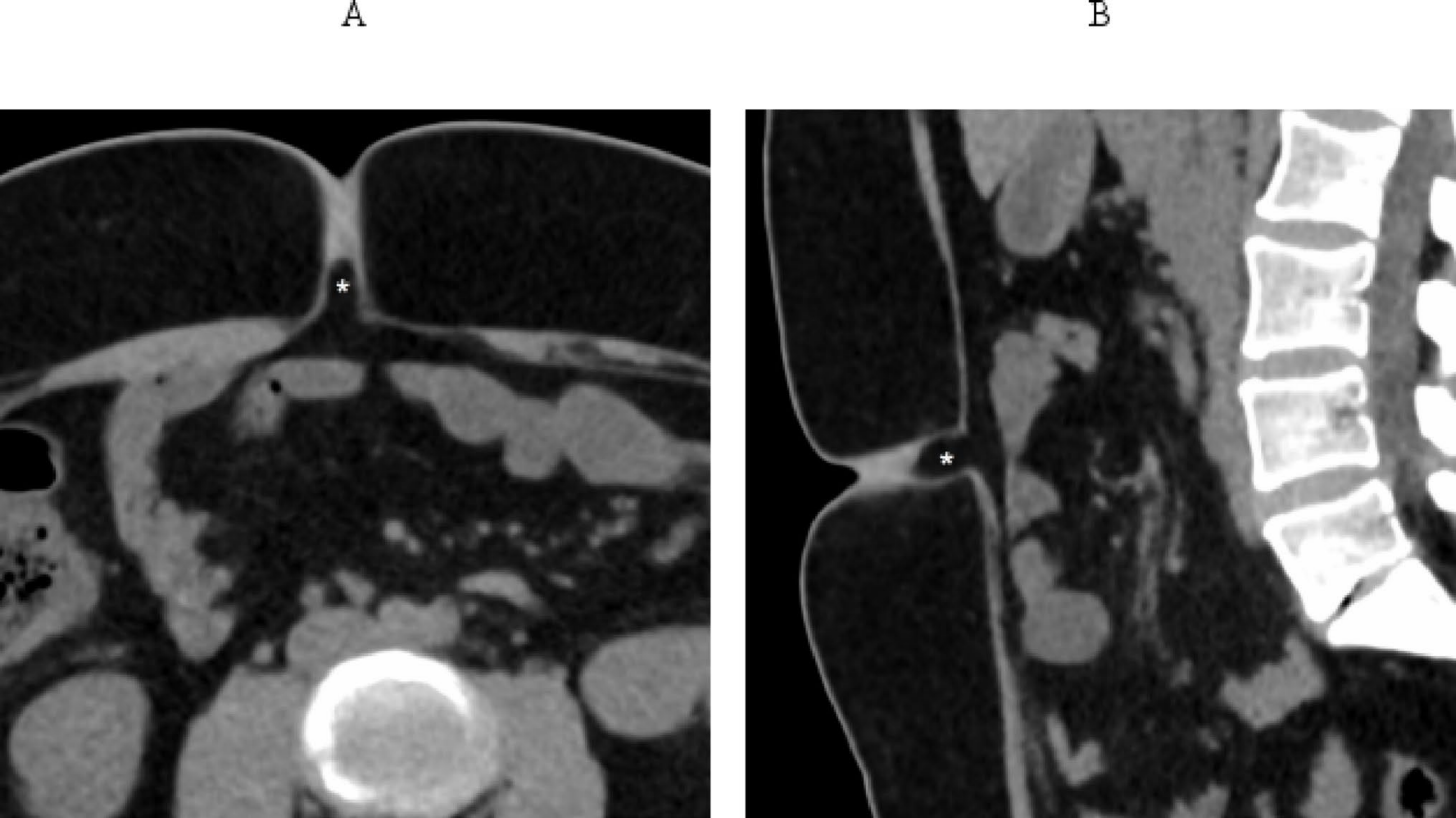
Axial (**A**) and sagittal (**B**) CT images demonstrate a fat-density tract (*) extending between the medial borders of the rectus abdominis muscles, posterior to the navel and anterior to the parietal peritoneum, without evidence of a peritoneal-lined sac or visceral herniation, increasingly labeled as an “umbilical hernia” in current radiology reports, however better described as the the umbilical canal

The widespread availability of abdominal CT, together with the global escalation of obesity, has transformed the routine visualization of the anterior abdominal wall. Among the most frequent incidental findings is a fat-density structure extending between the medial borders of the rectus abdominis muscles, posterior to the navel or umbilical scar and anterior to the parietal peritoneum. In contemporary radiology reports, this appearance is increasingly labeled as an “umbilical hernia” (Fig. 1) [1].

This terminology, while intuitively appealing, obscures a long-standing anatomical distinction described in the 19th and early 20th centuries: the umbilical canal, a potential preperitoneal space variably present between the posterior surface of the linea alba and a reinforcing lamella of the transversalis fascia, often containing preperitoneal fat and vestiges of the fetal umbilical structures [2, 3]. The rediscovery of this concept in the era of cross-sectional imaging invites a re-examination of how radiologic morphology is translated into clinical and surgical language.

The aim of this perspective article is threefold: (1) to revisit the historical and anatomical foundations that distinguish a true umbilical hernia from the umbilical canal, (2) to correlate these concepts with modern CT appearances, and (3) to examine how contemporary surgical risk metrics, increasingly derived from imaging, may be inappropriately extrapolated to a structure that does not conform to the geometric assumptions of a hernia.

## Historical and anatomical foundations

Classical surgical anatomy has consistently defined a hernia as a structural failure of the abdominal wall characterized by the protrusion of a peritoneal-lined sac through a fascial defect, containing omentum or bowel, and connected to the abdominal cavity by a recognizable and usually narrow neck [3–6]. Although historical in origin, these classical anatomical descriptions remain directly relevant to modern imaging interpretation because they establish the structural criteria that continue to define a true hernia in surgical and radiologic practice. The conceptual roots of this definition can be traced to antiquity. References to umbilical swellings appear in the Egyptian Papyrus of Ebers (circa 1552 B.C.), and the earliest formal medical descriptions are found in the writings of the Hindu physician Charaka (first century A.D. or earlier). Ancient Jewish medical traditions similarly recognized umbilical hernias and advocated predominantly conservative management.

In the Greco-Roman world, Celsus (first century A.D.) described techniques of elastic suturing for umbilical hernias, while Soranus (A.D. 98–117) proposed strapping methods, reflecting an early appreciation of reducibility and mechanical containment [4, 5]. A decisive conceptual advance was articulated by Albucasis (Abu al-Qasim al-Zahrawi) in the early eleventh century, whose Kitab al-Tasrif provided the first recorded description of operative repair of umbilical hernia [6]. Albucasis explicitly framed the condition as a protrusion of intra-abdominal contents, noting that “the navel becomes prominent… from a rupture of the peritoneum over the abdomen, so that omentum or intestine comes through it as in other ruptures,” and emphasizing hallmark clinical features such as reducibility with digital pressure, recurrence with exertion, and enlargement under conditions of increased intra-abdominal pressure. This sac-and-content paradigm firmly anchored umbilical hernia within the broader category of visceral herniation, rather than superficial or preperitoneal protrusions.

This conceptual lineage was refined in the late eighteenth and early nineteenth centuries by anatomists and surgeons working in the era of systematic dissection. In his seminal treatise, Scarpa emphasized that the hernial sac is “properly speaking, formed by the peritoneum,” and that true umbilical hernias invariably present a short, circular, and proportionally narrow neck. The sac, he noted, typically contains omentum and/or intestine, which may displace or obliterate the umbilical ligaments as the hernia enlarges [7].

Crucially, Scarpa also described a distinct and easily confounded entity: a small, firm mass of fat situated between the peritoneum and the aponeurotic fibers of the abdominal wall that may protrude through a fissure of the linea alba or into a structure that would later be recognized as the umbilical canal, externally mimicking an omental hernia. Through careful dissection, he demonstrated that these tumors lack a peritoneal-lined hernial sac and visceral content, consisting instead of a pedunculated continuation of preperitoneal fat, often in continuity with fat surrounding the obliterated umbilical vein (the ligamentum teres). He explicitly warned that such lesions may be clinically indistinguishable from small true hernias and may lead to unwarranted surgical intervention when misinterpreted [7].

A complementary anatomical framework was later provided by Cullen, synthesizing the work of Richet, Gauderon, and Stratz. Their dissections demonstrated marked variability in the relationship between the peritoneum, the transversalis fascia, and the navel. Richet described a fibrous lamella—the so-called umbilical fascia—reinforcing the peritoneum above the umbilical ring in some individuals and delimiting a space between this fascia and the posterior surface of the linea alba, which he termed the “umbilical canal.” This canal contains the fibrocellular remnants of the umbilical vein (ligamentum teres), obliterated umbilical arteries (medial umbilical ligament), and surrounding connective tissue, the meshes of which may be variably filled with preperitoneal fat [2].

During fetal development, the umbilical ring serves as a conduit for multiple structures connecting the embryo to the placenta, including the umbilical vein, paired umbilical arteries, the urachus, and the omphalomesenteric duct with its vessels [2]. After birth, these structures undergo progressive involution: the umbilical vein becomes the ligamentum teres of the liver, the umbilical arteries form the medial umbilical ligaments, and the urachus persists as the median umbilical ligament. Simultaneously, the umbilical ring is closed by fibrous and fascial remodeling of the linea alba and transversalis fascia. However, considerable variability exists in the remodeling of the umbilical ring. In some individuals, there is little or no separation between the parietal peritoneum and the posterior surface of the linea alba at the level of the umbilicus. In others, this developmental remodeling results in the formation of a potential preperitoneal space containing fibrocellular remnants of fetal structures surrounded by variable amounts of adipose tissue—corresponding to the umbilical canal [2]. This developmental continuum helps explain why the umbilical canal represents a variable anatomical configuration rather than a true fascial defect of the abdominal wall.

Importantly, Gauderon’s and Stratz’s dissections confirmed that a complete umbilical canal is not a constant anatomical feature and is predominantly observed in individuals with a greater volume of preperitoneal adipose tissue, particularly in obese subjects, while being absent or poorly developed in lean individuals, in whom the peritoneum may lie directly against the posterior aspect of the navel without any definable canal [2].

Together, these classical observations establish a clear anatomical and conceptual distinction between a true umbilical hernia—defined by a peritoneal sac, a fascial neck, and visceral content—and a umbilical canal, which represents a variable, habitus-dependent anatomical configuration rather than a defect of the abdominal wall. Importantly, the umbilical canal should be understood as a potential preperitoneal space formed by variable separation between fascial and peritoneal layers, rather than a true fascial defect of the abdominal wall, distinguishing it fundamentally from the structural failure that defines a hernia.

This anatomical distinction was later reinforced in early twentieth-century surgical pathology, as Moschcowitz emphasized that the clinical behavior and complication profile of abdominal wall hernias are determined by the presence of a peritoneal sac and a constricting fascial neck, rather than by superficial protrusions of fat or fibrous tissue, thereby bridging classical anatomy with emerging concepts of hernia pathophysiology [8].

## The CT era and semantic drift

**Fig. 2:**
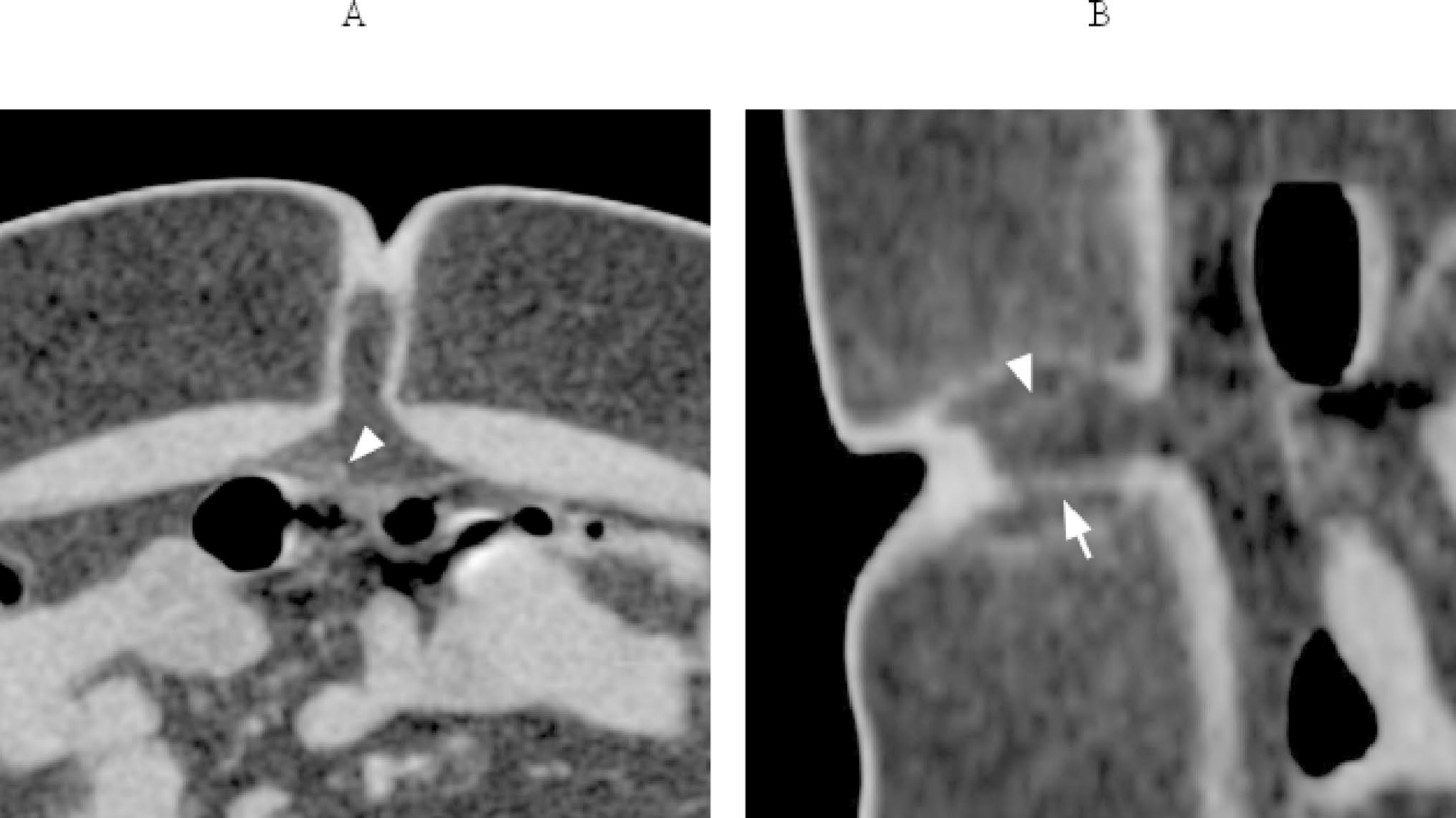
Magnified axial (**A**) and sagittal (**B**) CT view of the umbilical canal demonstrates linear soft-tissue structures within the preperitoneal fat tract, corresponding to the ligamentum teres (arrowhead) and the median umbilical ligament (arrow)

Cross-sectional imaging has transformed the visualization of the anterior abdominal wall. On axial and sagittal CT images, the umbilical canal typically appears as a funnel of fat-density tissue continuous with the preperitoneal compartment, extending between the medial borders of the rectus abdominis muscles and bounded by a distended linea alba. In some cases, linear soft-tissue densities corresponding to the median umbilical ligament (involuted umbilical arteries) and the ligamentum teres (obliterated umbilical vein) can be identified within or along the margins of this fatty tract (Fig. 2) [1, 2]. Importantly, this configuration is observed without a discrete peritoneal-lined sac.

This CT appearance closely mirrors the anatomical configuration of the umbilical canal described by Richet and later confirmed by Gauderon and Stratz: a variable, fat-containing channel posterior to the umbilical scar rather than a true herniation of intraperitoneal contents. The increased conspicuity of this structure in contemporary practice likely reflects both the near-universal use of CT and the greater volume of preperitoneal adipose tissue in modern patient populations, which accentuates a canal that may be nonapparent in lean individuals.

**Fig. 3:**
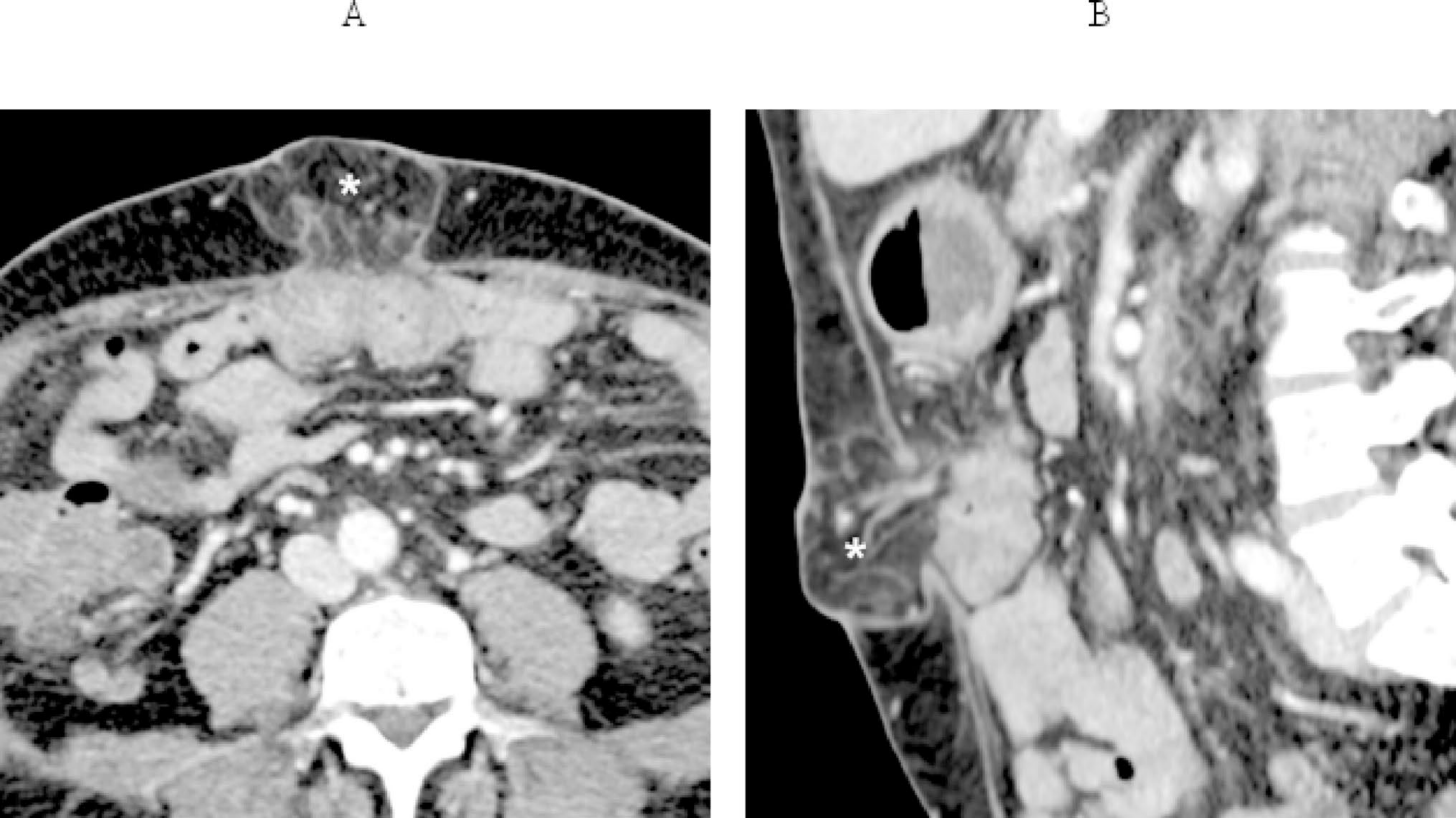
Axial (**A**) and sagittal (**B**) CT images demonstrate a true umbilical hernia with a peritoneal-lined sac containing omentum (*) protruding through a narrow fascial neck. The herniated omentum can be distinguished from preperitoneal fat by its direct intracavitary continuity, particularly by tracing its vascular structures as they remain contiguous with the intra-abdominal omental vessels. In contrast to the umbilical canal, embryologic remnants such as the median umbilical ligament and the ligamentum teres are not identifiable within the hernia sac

In contrast, a true umbilical hernia is characterized by a narrow fascial neck and a relatively wide peritoneal-lined sac containing visceral content, most commonly omentum with or without an associated bowel loop—frequently the transverse colon. When the sac contains only omentum, differentiation from preperitoneal fat can be achieved by demonstrating the intracavitary continuity of the omentum, particularly by tracing its vascular structures as they remain contiguous with the intra-abdominal omental vessels (Fig. 3). An additional distinguishing feature is the absence of identifiable embryologic remnants within the herniated fat, as the median umbilical ligament and the ligamentum teres are typically no longer visualized within the sac, in contrast to the umbilical canal configuration.

**Fig. 4:**
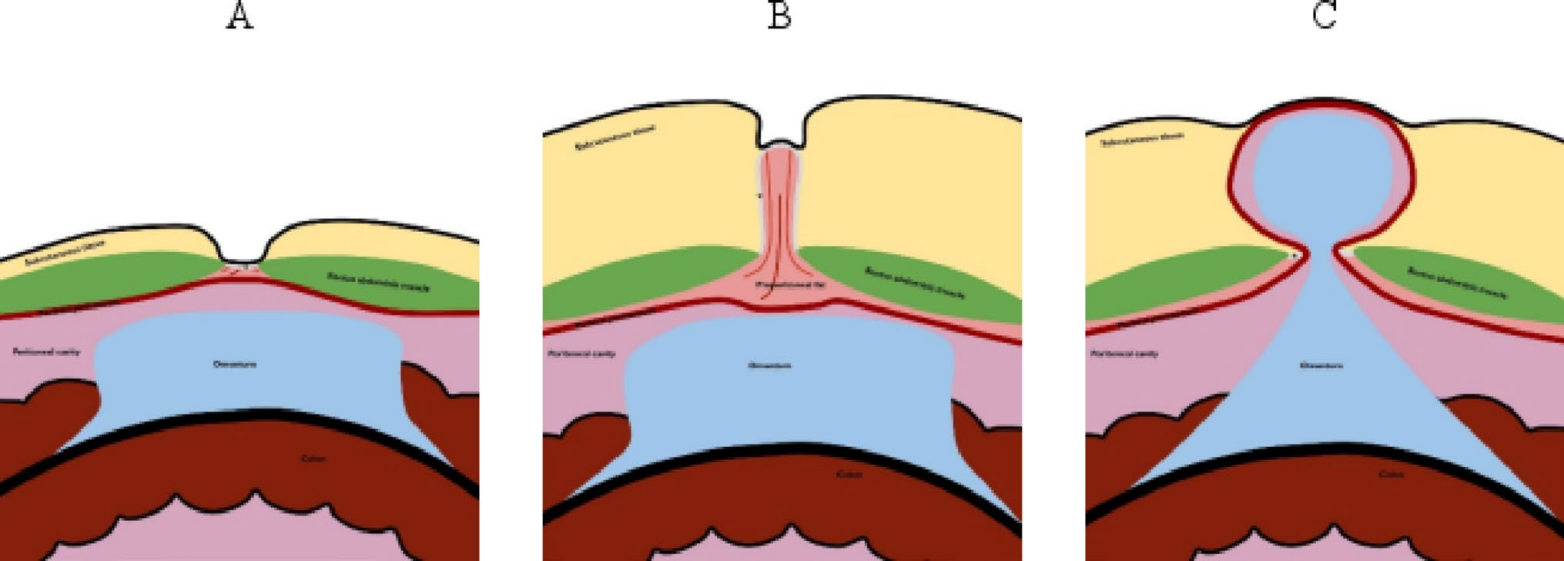
Schematic cross-sectional diagrams of the umbilical region illustrating the anatomical continuum between normal configuration, umbilical canal, and true umbilical hernia. **A** In a lean individual, the parietal peritoneum lies posterior to the linea alba (*) at the level of the navel, without a definable umbilical canal. **B** In an individual with increased adipose tissue, a potential space containing preperitoneal fat and fibrocellular remnants of the fetal umbilical structures is interposed between the navel and the parietal peritoneum, limited laterally by the linea alba (*), corresponding to the umbilical canal. **C** True umbilical hernia demonstrating protrusion of a peritoneal-lined sac containing omentum through a narrow fascial defect or neck (*)

From a CT perspective, the distinction between the umbilical canal and a true umbilical hernia can be established using a combination of morphologic criteria. The umbilical canal appears as a preperitoneal, fat-filled tract without a peritoneal-lined sac or discrete fascial defect, often demonstrating a tubular or gently funnel-shaped configuration with similar proximal and distal diameters. Embryologic remnants of the umbilical vessels may be identifiable within this space. In contrast, a true umbilical hernia demonstrates a focal fascial defect at the umbilical ring, a peritoneal-lined sac, and continuity of herniated content with the intraperitoneal cavity. The presence of a sac-and-neck configuration and visceral continuity are the most reliable CT features distinguishing true herniation from preperitoneal fat interposition. To facilitate practical application in radiology reporting, the key CT features distinguishing these entities are summarized in Table 1, Fig. 4.

**Table 1:** CT criteria distinguishing the umbilical canal from a true umbilical hernia

Imaging features	Umbilical canal	True umbilical hernia
Peritoneal-lined sac	Absent	Present
Content	Preperitoneal fat	Omentum ± bowel loop
Morphology	Tubular or funnel-shaped fat tract (similar proximal and distal diameters)	Sac-and-neck configuration (sac diameter > neck diameter)
Continuity with abdominal cavity	Absent (intact parietal peritoneum separating the canal and the peritoneal cavity)	Present (omental vessels raceable to intraperitoneal omentum)
Umbilical ligaments (median ligament, ligamentum teres)	May be visible	Typically not identifiable within the sac

## Clinical implications for radiology reporting

From a radiologic standpoint, the distinction between a true hernia and the umbilical canal is not merely academic. Labeling the umbilical canal as a hernia may prompt surgical referral, patient anxiety, additional imaging, and potentially unnecessary interventions. Because incidental anterior abdominal wall findings are frequently encountered on CT performed for unrelated indications, imprecise terminology may unintentionally medicalize a habitus-dependent anatomical configuration that is often asymptomatic and structurally stable.

Contemporary practice guidelines emphasize defect size, symptomatology, and patient-specific risk factors when considering repair of umbilical and ventral hernias; however, these recommendations are largely predicated on the presence of a true hernia defect with a peritoneal sac and a measurable fascial neck, a scenario that does not explicitly encompass the preperitoneal, fat-filled configuration of the umbilical canal [9].

As imaging has assumed an increasingly prominent role in surgical triage and risk assessment of ventral and umbilical hernias, radiologic morphology is now routinely translated into clinical decision-making frameworks. Within this context, the hernia-to-neck ratio has emerged as a reproducible morphologic predictor of incarceration and emergent repair, demonstrating that clinical relevance rises sharply when the maximal diameter of the hernia sac exceeds that of the fascial neck by a factor of approximately 2.5 [10, 11]. In practical terms, this threshold implies that a hernia acquires meaningful clinical risk primarily when a large, expansile sac is constrained by a relatively narrow neck.

When this concept is extrapolated to the umbilical canal, a fundamentally different geometry becomes apparent. On CT, the diameter of the canal measured proximally near the parietal peritoneum and the maximal diameter measured distally near the umbilical scar are typically similar, reflecting a tubular or gently funnel-shaped fat-filled tract rather than a sac-and-neck configuration. By hernia-to-neck ratio-based criteria, such a morphology corresponds to a low-risk profile, even under the most liberal application of the term “hernia.”

Beyond risk stratification, consistent terminology in radiology reports may influence interdisciplinary communication and longitudinal patient management. Describing this configuration as an umbilical canal acknowledges its anatomical basis while avoiding implicit assumptions regarding surgical relevance. Conversely, labeling the finding as a hernia may lead to variability in interpretation among clinicians, particularly when imaging findings are discordant with physical examination or symptoms.

A more anatomically precise description—such as “umbilical canal with preperitoneal fat interposition, without evidence of a true hernia sac or visceral herniation at rest”—may better reflect the underlying structure while preserving clinical neutrality. This approach aligns with the traditional definition of hernia and acknowledges the broad variability of normal and near-normal umbilical anatomy described in classical dissections.

## Unresolved questions and future directions

Several clinically relevant uncertainties remain regarding the imaging and clinical significance of the umbilical canal. First, it is unknown whether the mere presence or enlargement of the umbilical canal—particularly beyond the historically cited threshold of approximately 10 mm—constitutes a risk factor for the future development of a true umbilical hernia. The available anatomical literature suggests that the canal reflects variability in preperitoneal tissue distribution rather than a structural defect, but longitudinal imaging data addressing this question are lacking.

Second, the extent to which the appearance of the umbilical canal varies with body habitus, age, or sex has not been systematically investigated. Classical anatomical observations and modern imaging experience suggest that the canal may be more conspicuous in individuals with increased preperitoneal adipose tissue, particularly in obese patients, whereas it may be absent or inapparent in lean individuals. However, these observations remain largely descriptive and have not been validated in dedicated imaging studies.

Third, routine CT examinations are performed in the dorsal decubitus and at rest, conditions that may underestimate dynamic anterior abdominal wall changes. It remains unclear whether physiologic maneuvers such as Valsalva could, in selected individuals, demonstrate transient protrusion of intraperitoneal content into the limits of the umbilical canal, potentially representing a continuum between normal anatomy, preperitoneal fat interposition, and true herniation.

Finally, the natural history of the umbilical canal remains undefined. Whether this configuration represents a benign anatomical variant, a habitus-dependent imaging finding, or a potential pre-hernial state is unknown. Prospective imaging studies integrating dynamic assessment, anthropometric data, and longitudinal follow-up will be necessary to clarify these questions and to determine the clinical significance—if any—of the umbilical canal in modern radiology practice.

## Conclusion

The rediscovery of the umbilical canal concept provides a valuable anatomical framework for interpreting a common CT finding in the modern imaging era. Recognizing this structure as distinct from a true umbilical hernia may improve terminological precision, enhance interdisciplinary communication, and help avoid the unintended consequences of overdiagnosis.

## Data Availability

No datasets were generated or analysed during the current study.
